# The miR-206/133b cluster is dispensable for development, survival and regeneration of skeletal muscle

**DOI:** 10.1186/s13395-014-0023-5

**Published:** 2014-12-12

**Authors:** Thomas Boettger, Stas Wüst, Hendrik Nolte, Thomas Braun

**Affiliations:** Department of Cardiac Development and Remodelling, Max-Planck-Institute for Heart and Lung Research, Ludwigstraße 43, 61231 Bad Nauheim, Germany

**Keywords:** miR-206, miR-133b, miR-1, MDX, Muscle regeneration, Pax7

## Abstract

**Background:**

Three different gene clusters code for the muscle-specific miRNAs miR-206, miR-1 and miR-133a/b. The two miR-1/133a clusters generate identical mature miR-1 and miR-133a miRNAs in heart and skeletal muscle, while the cognate miR-206/133b cluster is exclusively expressed in skeletal muscle. Since sequences of the miRNAs miR-133a and miR-133b are almost identical, it seems likely that they share potential targets. Similarly, miR-1 and miR-206 are structurally related and contain identical seed sequences important for miRNA-target recognition. In the past, different functions of these miRNAs were suggested for development, function and regeneration of skeletal muscle using different *in vivo* and *in vitro* models; however, mutants lacking the complete miR-206/133b cluster, which generates a single pri-miRNA constituting a functional unit, have not been analyzed.

**Methods:**

We generated miR-206/133b knock-out mice and analyzed these mice morphologically; at the transcriptome and proteome level to elucidate the contribution of this miRNA cluster for skeletal muscle development, differentiation, regeneration *in vivo*; and by systematic analysis. In addition, we studied the consequences of a genetic loss of miR-206/133b for expression of Pax7 and satellite cell differentiation *in vitro*.

**Results:**

Deletion of the miR-206/133b cluster did not reveal any obvious essential function of the miRNA-cluster for development and differentiation of skeletal muscle. Careful examination of skeletal muscles of miR-206/133b mutants revealed no structural alterations or molecular changes at the transcriptome and proteome level. In contrast to previous studies, deletion of the miR-206/133b cluster did not impair regeneration of skeletal muscle in *mdx* mice. Likewise, differentiation of miR-206/133b deficient satellite cells *in vitro* was unaffected and no change in Pax7 protein concentration was apparent.

**Conclusions:**

We conclude that the miR-206/133b cluster is dispensable for development, function and regeneration of skeletal muscle, probably due to overlapping functions of the related miR-1/133a clusters, which are strongly expressed in skeletal muscle. We reason that the miR-206/133b cluster alone is not an essential regulator of skeletal muscle regeneration, although more subtle functions might exist that are not apparent under laboratory conditions.

## Background

miRNAs regulate protein expression at the post-transcriptional level by decreasing transcript abundance or inhibiting protein translation. The miRNAs miR-1, miR-206 and miR-133a/b are specifically expressed in striated muscle. The mouse genome contains two miR-1/133a gene clusters located on chromosome 2 and 18, giving rise to identical mature miR-1 and miR-133a miRNAs while the structurally related miR-206/133b cluster is located on mouse chromosome 1. The mature miR-133b differs in only one nucleotide from miR-133a and the primary sequence of miR-206 is highly related to miR-1. Importantly, miR-1 and miR-206 do not differ in the seed sequence that is assumed to determine target specificity of miRNAs [1]. Deletion of both miR-1/133a clusters, which are expressed in heart and all skeletal muscles, results in early embryonic lethality due to defects in heart development caused by the failure to restrict myocardin expression and concomitant upregulation of smooth muscle genes [2]. Deletion of both miR-1 copies leads to cardiac defects with partially penetrant neonatal lethality [3] but did not cause a major skeletal muscle phenotype, which was attributed to the remaining expression of miR-206 in skeletal muscle. Deletion of both miR-133a copies affects cardiomyocyte proliferation and heart physiology [4] and results in a centronuclear skeletal myopathy and a shift of muscle fiber identity from glycolytic to oxidative muscle fibers in the soleus muscle [5].

The miR-206/133b cluster is not expressed in the heart, but its expression is confined to developing skeletal muscle. In adult muscles miR-206/133b are preferentially found in slow myofibers. Expression of miR-206/133b is controlled by a network of myogenic regulatory genes [6,7], including MyoD, which binds to the miR-206/133b locus in C2C12 cells [8]. The miR-206/133b locus not only encodes the miRNAs miR-206 and miR-133b, but also the long non-coding RNA linc-MD1, which is expressed during muscle development similar to miR-206 and miR-133b and assumed to act as a competing endogenous RNA or miRNA decoy [9]. Genomic deletion of miR-206 did not cause an obvious clinical phenotype in mice, although it was determined that miR-206 is required for re-innervation of muscle tissue in mice with mutant Sod1 [10]. It was proposed that miR-1/206 repress Hdac4 [11], which in turn might regulate genes involved in controlling muscle-derived signals that enhance synapse formation under pathological conditions [10]. More recently, miR-206 was claimed to act as a modifier of muscular dystrophy [12]. Deletion of miR-206 in the mdx mouse model of Duchenne muscular dystrophy [13,14] led to increased lethality in compound mutants and accumulation of degenerated muscle fibers. Surprisingly, no defects in muscle innervation were observed after the loss of miR-206, although muscle fibers are continuously lost and replaced in mdx mice. The dramatic enhancement of muscle dystrophy in miR-206/mdx compound mutants was attributed to impaired differentiation of myofibers due to the up-regulation of Pax7, which contains identical binding sites for miR-206 and miR-1 in its 3′ untranslated region (UTR) [12]. The presence of miR-206 target sites in Pax7 did also motivate other researchers to investigate a regulation of Pax7 by miR-206, revealing that overexpression or *knock-down* of miR-206 can affect Pax7 levels and thereby muscle differentiation *in vitro* [15,16].

Here, we analyzed mice with a targeted deletion of the miR-206/133b cluster. miR-206/133b are processed from a common precursor and thus might be regarded as a functional unit similar to the miR-1/133a clusters [2]. Concomitant loss of miR-206 and miR-133b neither leads to an obvious clinical phenotype or causes detectable molecular changes in skeletal muscles nor impairs muscle regeneration in the MDX mouse model of muscular dystrophy. Surprisingly, lack of miR-206/133b and the miR-133 decoy, contained in the third exon of linc-MD1, did not have obvious effects on satellite cell proliferation and differentiation. Our findings differ from a previous analysis of miR-206 mutants, which might suggest that other products processed from the primary miR-206/133b transcript balance effects of miR-206 *in vivo*.

## Methods

### Ethics statement

Animal maintenance and animal experiments were in accordance with German animal protection laws and were approved by the local governmental animal protection committee (Regierungspraesidium Darmstadt, Federal State of Hessen).

### Deletion of miR-206/133b

The miR-206/133b coding region was deleted from the mouse genome using a vector containing genomic sequences flanking the miRNA miR-206/133b gene and a PGK-neomycin selection cassette. The short arm (2.3 kb) used for recombination encompassed the miR-206 5′ flanking region from TTAGGCATATAAAGTTTGCACGACC to GATATAAAGAAGCATGTGGCCTGGG). The long arm of the targeting vector consisted of a 5.9 kb NcoI fragment directly downstream of miR-133b. A DTA selection cassette was used for negative selection against nonhomologous insertion of the vector into the genome. Homologous recombination in mouse embryonic stem (ES) cells was ascertained by Southern blot analysis using a SphI digest and a 453 bp 5′ probe amplified by the oligonucleotides (TAAGTCCTGATGCTTCTCAATACCC; GTTGATAAAGAAACTGTGTGTTACG), resulting in an 8.4-kb WT and an 11.8-kb knock-out signal. Although the inserted β-galactosidase coding (lacZ) cassette contained an IRES sequence, no β-galactosidase was expressed from the engineered locus. Genotyping of mdx mice followed the protocol described by Shin *et al*. [17]. Primary sequences of mature miRNAs were derived from mirBase20 (MIMAT0000239, MIMAT0000123, MIMAT0000769 and MIMAT0000145) [18].

### RNAse protection assay and northern blot

Total RNA was isolated from M. soleus of PBS perfused animals using the TRIZOL method (Invitrogen, Thermo Fisher Scientific, Darmstadt, Germany). The RNase protection assay was performed using the mirVana Probe construction Kit (Ambion, Thermo Fisher Scientific, Darmstadt, Germany; Cat#1550) using oligos (miR-206: TGGAATGTAAGGAAGTGTGTGG, miR-145: GTCCAGTTTTCCCAGGAATCCCTTTTTTTT) together with the mirVana miRNA Detection Kit (Ambion#1552) following the manufacturer’s instructions. A polyT at the control oligo was added to allow size resolution of the control oligo. The sequence CCTGTCTC was added to the 3′ end of the oligos to allow binding of the Ambion T7-Promoter Primer. Amersham α-^32^P UTP (GE Healthcare, Freiburg, Germany; PB20383, 800 Ci/mmol) was used for labeling. Probes were purified using a 15% Invitrogen TBE-Urea Gel (EC68855BOX), the gel was exposed to a Kodak X-ray film, labeled probes were excised und eluted 30 min in elution buffer. 5 μg of total RNA isolated from WT, heterozygous or KO-muscle or yeast-RNA (Roche, Mannheim, Germany), respectively was hybridized at 42°C overnight with approximately 10^4^ cpm of probe in hybridization buffer in a total volume of 20 μl. Samples were analyzed using 15% TBE-Urea gels after RNase A/T1 digestion. Signals were detected using Kodak X-Ray film. Northern blot analysis was performed as previously described [19]. Antisense oligonucleotides specific for miR-206 (CCACACACTTCCTTACATTCCA), miR-1 (ATACATACTTCTTTACATTCCA) and U6 (ATATGGAACGCTTCACGAATT) were used.

### Microarray analysis, quantitative reverse transcription PCR and small RNA sequencing

RNA quality was analyzed with the Agilent Bioanalyzer and the RNA 6000 Nano Kit (Agilent Technologies, Santa Clara, CA). For RNA expression analysis, the Affymetrix GeneChip MouseGenome 430 2.0 Array was used, employing the one-cycle target labeling protocol. Data were analyzed using the Affymetrix expression console and the RMA algorithm. Microarray data have been submitted to Arrayexpress (Acc#E-MTAB-2439). TaqMan MicroRNA Expression Assays and the Applied Biosystems StepOnePlus system were used to quantify miR-206 and miR-1 expression. The RT reaction was done using the miR-206 and the U6 specific primer with the Taqman MicroRNA Reverse Transcription Kit (#4366596). qPCR assays were performed with three independent samples each with Taqman Universal PCR Master Mix (#4324018), FAM labeled miR-206 or miR-1 assay (#000510; #002222) and VIC labeled U6 assay (#001973) for normalization. Relative expression was calculated using the ∆∆Ct method. RNA isolated from a pool of M. soleus of five male C57Bl6 mice was sequenced using IonTorrent sequencing and analyzed as described [20]. Data were submitted to the NCBI’s Gene Expression Omnibus (Acc#GSE63342).

### Whole-mount *in situ* hybridization

For whole moun*t in-situ* hybridization, a myogenin cDNA corresponding to 307 to 1393 bp of ENSMUST00000027730 was used to synthesize a DIG-labeled antisense probe by T7-RNA-polymerase. E10.5 embryos were isolated and genotyped after mating of heterozygous parents.

### Fiber type determination, histology

For histological analysis, muscle tissue was perfused *in situ* with 4% PFA, dissected, washed in PBS, dehydrated and embedded in paraffin, followed by preparation of 10 μm sections, deparaffinization and hydration in distilled water. Von Kossa staining was performed by incubating slides with 1% silver nitrate solution and exposed to light for 30 min. Slides were washed two times for 3 min in H_2_O and incubated in 5% sodium thiosulfate solution for 5 min. Thereafter, slides were washed two times in H_2_O, followed by counterstaining for 7 min in 0.1% EosinG (Merck, Darmstadt, Germany) with 0.05% acetic acid. Slides were dehydrated and mounted using Entellan. For Sirius red staining, slides were stained in Weigerts Iron-Hematoxylin solution (Sigma, Munich, Germany; Cat#HT1079) for 8 min, dipped twice in distilled water and stained one hour in 0.1% Direct Red 80 (Sigma#365548)/saturated picric acid. Subsequently, slides were washed using 0.5% acetic acid, dipped in distilled water, dehydrated and mounted using Entellan.

For determination and quantification of fiber types in the muscle tissue, tissue was perfused *in situ* with 4% PFA, isolated and incubated in 15% and 30% sucrose/PBS for 2 hours and overnight, respectively. Tissue was frozen on dry ice and cryotome-sectioned. Sections were mounted on Superfrost slides. Tissue was dried and subsequently treated with 4% PFA/0.1% sodium desoxycholate/0.02% NP-40 for 5 min, washed 3 times with PBS for 5 min, blocked in 2% FCS, 0.5% NP-40/PBS for 1 h and then incubated with monoclonal anti-myosin (skeletal, slow; Sigma, M8421) in 2% FCS/0.5% NP-40/PBS overnight at 4°C. Slides were washed three times with PBS for 5 min and then incubated with biotinylated secondary antibody (Vector Labs, Burlingame, CA; BA-1400) for 2 h at RT and further processed according to the Vectastain Elite ABC Kit.

### Satellite cell isolation and culture

Satellite cells were isolated from the hind leg muscles of approximately 20 to 25-week-old wildtype and miR-206/133b knock-out animals by using the skeletal muscle dissociation kit (Miltenyi Biotech, Bergisch Gladbach, Germany, 130-098-305) and enriched by the satellite cell isolation Kit, mouse (Miltenyi Biotech, 130-104-268) according to the manufacturer’s instructions. Isolated cells were plated on gelatin-coated μclear 96-well plates (Sigma#M0562). Satellite cells were grown in proliferation medium (40% DMEM, 40% Ham F-10; 20% FCS; Pen/Strep; 2.5 ng/ml human FGF-2, Miltenyi Biotech#130-093-840) for 3 days, followed by switch to differentiation medium (DMEM, 2% horse serum, and Pen/Strep). Cells were incubated for 5 min in fixative (4% PFA/PBS, 0.1% sodium desoxycholate, 0.2% NP-40), washed 3 times in PBS, blocked in carrier (PBS, 5% BSA, 0.5% NP-40) for 1 h and then incubated with MF 20 supernatant in carrier (1:100, DSHB, Iowa City, Iowa). Secondary antibody was goat anti-mouse IgG1 Alexa 488 (1:2000, Life technologies).

### Quantitative proteomics

Soleus muscle was dissected from adult ^13^C_6_Lys-labeled (SILAC) WT and from nonlabeled WT and miR-206/133b mutant littermates. Protein extracts were processed and mass spectrometry was performed as described previously [19,21]. Briefly, 30 μg protein extracts of soleus muscle of labeled and the respective unlabeled muscle were combined, size-fractionated into 10 fractions using SDS-PAGE, and the proteins were digested in-gel using the endoproteinase LysC. After digestion peptides were eluted from gel-fractions using an acetonitrile gradient. Peptides were desalted prior to LC-MS/MS analysis and loaded onto a 15 cm in-house packed column (75 μm ID, C18 Beads 3 μm diameter, Dr. Maisch, Germany) and eluted by an linear increase of buffer B within a binary buffer system: A) 0.5% acetic acid and B) 80% ACN, 0.5% acetic acid. The flow-rate was set to 200 nL/min and gradient time to 150 min. The Agilent 1200 nanoflow HPLC system was coupled to an LQT-Orbitrap XL (Thermo Scientific) via an electrospray ionization source (Thermo Fisher Scientific). The mass spectrometer was operated in the data-dependent mode to automatically measure full MS scans and MS/MS spectra. In detail, survey scans were measured after accumulation of 1E6 ions at a resolution of 60,000 and subsequently isolation and fragmentation (CID, 35% norm. collision energy, target value: 5,000) of the top five intense peaks was performed in the linear ion trap. Peptides were identified by correlation of acquired MS/MS spectra against the mouse IPI (ftp://ftp.ebi.ac.uk/pub/databases/IPI) using MaxQuant software [22]. A FDR cutoff of 1% was set on the protein and peptide level. Two mis-cleavages were allowed. Amidomethylation at cysteine residues was set as fixed modification, while oxidation on methione residues and acetylation at the N-term of proteins were defined as variable modifications. Quantification of proteins was performed by MaxQuant software. Only peptides with a minimal length of six amino acids and at least two ratio counts for SILAC pairs were used for quantification. Using SILAC as an internal standard, ratios miR-206/133b KO/^13^C_6_Lys-WT and ^13^C_6_Lys-WT/WT were obtained and were used for calculation of the direct KO/WT ratios for relative protein abundance.

### Western blot

For western-blot analysis of embryonic tissue, the trunk of E10.5 embryos posterior of the otic placode was used. A total of 10 μg of protein extract were loaded onto a 9% SDS-PAGE and blotted onto nitrocellulose membranes. The following antibodies were used: anti-Pax7 (supernatant of DSHB#P3U1), anti-pan-actin (1:1000, CST, Frankfurt, Germany; Cat#4968), anti-GAPDH (1:1000, CST; Cat#2118), secondary antibodies were anti-mouse-HRP (1:1000, eBioscience, Affymetrix, Frankfurt Germany, Cat#18-8816-31) and anti-rabbit-HRP (1:5000, Pierce, Thermo Fisher Scientific, Darmstadt, Germany, Cat#1858415).

### Statistics

Data are reported as mean ± SEM. Differences between groups were tested for statistical significance using the unpaired Student’s. For microarray experiments the median of groups was used and statistical analysis using an unpaired t-test was performed using DNAStar Arraystar 11 software (annotation na33). The Benjamini-Hochberg FDR correction [23] was used to control the multiple hypothesis testing in case of the microarray experiments.

## Results

### Deletion of the miR-206/133b cluster does not alter skeletal muscle morphology

**Figure 1:**
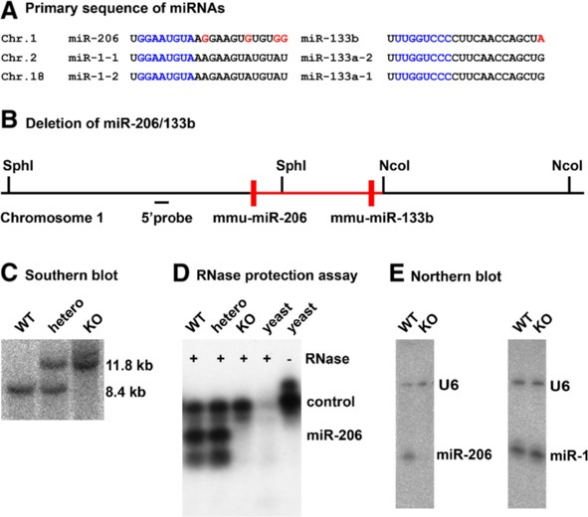
**Generation of miR-206/133b knock-out mice.** The miRNAs miR-206, miR-1 and 133a/b are encoded in three clusters on mouse chromosome 1, 2 and 18. The primary sequence **(A)** of mature miR-1/133a encoded on chromosome 2 and 18 is identical, miR-206 differs in four bases from miR-1, and miR-133b differs in one base from miR-133a (red). The seed sequence, which is instrumental for target binding of miRNAs, is identical between the corresponding miRNAs (blue). Schematic representation of the miR-206/133b genomic region **(B)** with deleted parts indicated (red). Southern blot analysis **(C)** of genomic DNA isolated from wildtype (WT), heterozygous, and homozygous mutant animals proves deletion of the miR-206/133b locus. An SphI digest of genomic DNA and a probe hybridizing 5′ prime of the targeting vector were used to monitor targeting of the miR-206/133b locus by homologous recombination. RNase protection assay **(D)** and northern blot **(E)** analysis demonstrates the loss of miR-206 in the soleus muscle of knock-out animals. Expression of miR-1 is not changed in soleus muscle of miR-206/133b knock-out mice.

**Figure 2:**
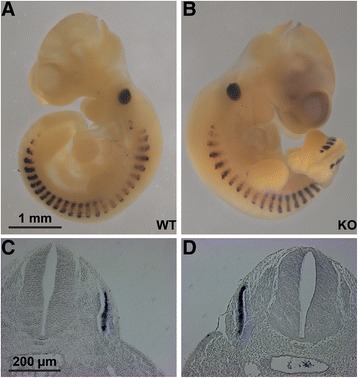
**Deletion of miR-206/133b does not impair myogenesis during somite development.** miR-206/133b knock-out animals are born with the expected Mendelian distribution (see text), whole mount *in situ* hybridization indicates normal myogenin expression in wildtype (WT; **A,** **C**) and knock-out embryos (KO; **B,** **D**) at embryonic day 10.5. Sections (**C,** **D**) are at the level of the forelimbs.

To analyze the function of the miR-206/133b miRNA cluster we deleted the coding region of the miR-206/133b gene on mouse chromosome 1 by homologous recombination (Figure 1A, B). The targeting strategy also removed the last exon of the ncRNA linc-MD1 including the putative miR-133a/b binding site in linc-MD1 [9], which is identical to the miR-133b* sequence. Neither the putative miR-135 binding sites in the second exon of linc-MD1, nor the described distal or proximal regulatory elements [9], identified by myogenin or myoD ChIP-seq approaches in C2C12 myocytes [8,24], were affected by the targeting strategy. Targeted deletion of the miR-206/133b coding region was validated by southern blot analysis (Figure 1C). Viable knock-out mice were obtained by breeding of heterozygous animals. Deletion of the miR-206/133b cluster did not impair embryonic or early postnatal survival of homozygous animals (Table 1; mdx^Y/+^ mice). To exclude a function of miR-206/133b in early development that might be compensated during later stages we analyzed the spatial distribution of myogenin transcripts at the embryonic stage E10.5. No differences in somite morphology and myogenin expression were detected (Figure 2). Development of body weights and survival rate of miR-206/133b knock-out mice was indistinguishable from WT animals. RNase protection analysis as well as northern blot analysis using RNA isolated from hind limb muscle confirmed the absence of miR-206 in homozygous mutant animals (Figure 1D, E). Due to the presence of miR-133a in skeletal muscles, which differs by only one nucleotide from miR-133b, it was not possible to demonstrate the absence of mature miR-133b in homozygous mutant animals.

**Table 1:** **Normal Mendelian distribution of offspring at the time of weaning**

**Genotype**	**N**	**% observed**	**Expected**
miR-206/133b^+/+^ mdx^y/+^	29	13.0%	12.5%
miR-206/133b^+/+^ mdx^y/-^	25	11.2%	12.5%
miR-206/133b^+/−^ mdx^y/+^	57	25.6%	25.0%
miR-206/133b^+/−^ mdx^y/-^	41	18.4%	25.0%
miR-206/133b^−/−^ mdx^y/+ (a)^	35	15.7%	12.5%
miR-206/133b^−/−^ mdx^y/- (b)^	36	16.1%	12.5%
**Total**	**223**	**100.0%**	**100.0%**

Deletion of the miR-206/133b cluster does not lead to abnormalities in the Mendelian distribution of mutant animals ^(a)^ at the time of weaning. Additional mdx mutation of the dystrophin gene does not lead to further lethality in double mutant animals ^(b)^. Distribution of male offspring after mating of male miR-206/133b^+/−^ mdx^y/+^ or miR-206/133b^+/−^ mdx^+/+^ to female miR-206/133b^+/−^ mdx^+/−^ is presented.

**Figure 3:**
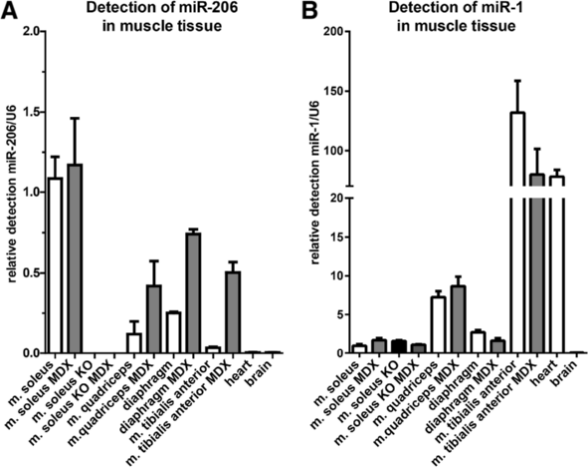
**Expression of miR-206 is confined to skeletal muscle.** Expression of miR-206 **(A)** is detected by quantitative reverse transcription PCR (qRT-PCR) in tissues isolated from wildtype (WT) (open bars), miR-206/133b knock-out and mdx^y/-^ (gray bars) mice. Expression of miR-206 is detected in skeletal muscle of the WT mice. miR-206 is not detected in muscles isolated from knock-out mice, confirming the loss of miR-206 in the knock-out tissue and specificity of the Taqman assay. miR-206 was at the qRT-PCR detection limit in heart and brain of WT mice. In WT mice, miR-206 was strongly expressed in the soleus muscle, which has a high content of type 1/oxidative skeletal muscle fibers. A lower amount of miR-206 was detected in the diaphragm, as well as in other skeletal muscles. In mdx mice, the expression of miR-206 was not increased in soleus muscle. Increased miR-206 expression was detected in the diaphragm and other skeletal muscles. Expression of miR-1 **(B)** was also analyzed in the respective muscles and appeared unchanged in the soleus muscle of miR-206/133b knock-out mice (black bar). *Mdx* mutation did not cause significant changes in the expression of miR-1. Samples were isolated from ≥3 different animals each, and qRT-PCR was performed in triplicate for each of the samples.

**Figure 4:**
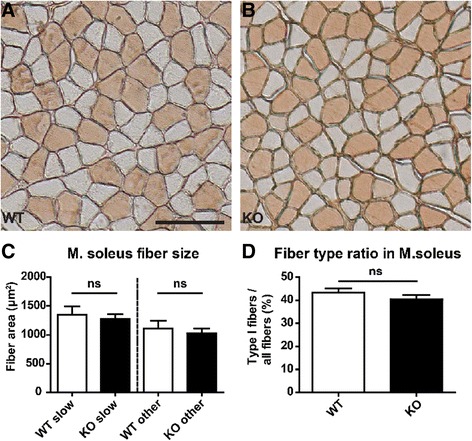
**No alterations in fiber type identity and fibers size in miR-206/133b knock-out mice.** Sections of the soleus muscle isolated from adult wildtype (WT) **(A)** and mutant mice **(B)** were stained for markers of slow myofibers. No changes in fiber size of slow and fast muscle fibers were detected (**C**; n ≥8 per group, 30 fibers per animals). The ratio of slow/fast muscle fibers was unchanged in the soleus muscle of knock-out compared to WT mice **(D)**. The scale bar in A corresponds to 100 μm in **A** and **B**.

The miR-206/133b cluster is predominantly expressed in muscles with a high content of oxidative type I myofibers such as the soleus muscle and less abundant in muscles with only few oxidative fibers (Figure 3A). Analysis of published small RNA sequencing results [25] reveals that miR-206 is much less abundant in skeletal muscle than the related miR-1 (GSM539875; 20124 miR-206 versus 745064 miR-1 tags per million miRNA tags). Small RNA sequencing of mouse soleus muscle revealed that even in the muscle with highest expression of miR-206 miR-1 is by far the most abundant miRNA (GSE63342; 30812 miR-206 versus 281944 miR-1 tags per million miRNA tags). The expression of miR-1 was not significantly altered after loss of miR-206 (Figure 3B, M. soleus samples). Histological examination of M. soleus did not reveal obvious morphological alterations. We detected no changes in fiber size, fiber type distribution (Figure 4) or localization of myofiber nuclei. Similar results were obtained for other skeletal muscles (data not shown).

### miR-206/133b mutant skeletal muscles do not show significant molecular abnormalities

**Figure 5:**
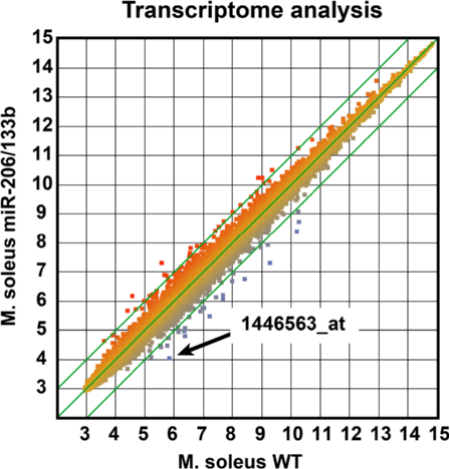
**No transcriptional changes in the soleus muscle of miR-206/133b knock-out (KO) mice.** The scatter blot of the median of the log2 signals of wildtype (WT) (n = 9) versus KO (n = 8) indicates only few genes with expression changes more than twofold (green lines). The probe set 1446563_at detects RNA originating from the genome between miR-206 and miR-133b most likely the pri-miRNA of these miRNAs. The weak signal for this probe set in WT samples is reduced to the detection limit in the KO samples. Only a few of the detected changes in gene expression are significant after statistical analysis using Student’s t-test. None of these changes could be verified by quantitative reverse transcription PCR (qRT-PCR) or analysis of protein expression. After false discovery rate correction by the Benjamini-Hochberg algorithm, only one gene directly downstream of the manipulated genomic locus significantly changed expression.

To investigate whether the loss of expression of miR-206/133b leads to changes of gene expression levels we performed Affymetrix GeneChip analysis using RNA isolated from M. soleus and mouse genome 430 2.0 arrays (n = 9 WT, n = 8 KO). Only few and subtle changes were detected by this type of analysis (Figure 5). The strongly and significantly downregulated probe set 1446563_at detects RNA transcribed from the miR-206/133b locus with the probes binding between the both miRNAs but not within the known linc-MD1 exons. This finding additionally confirms the successful knock-out strategy and suggests that this probe set detects the common miR-206/133b precursor RNA. The only gene that was significantly (2.5-fold) upregulated after false discovery rate correction [23] was IL-17a, which is located downstream of the miR-206/133b locus, suggesting a cis-regulatory mechanism caused by manipulation of the neighboring miRNA locus. No other gene was significantly deregulated in soleus muscle. In particular, we did not observe up-regulation of predicted miR-206 or miR-133b target genes.

**Figure 6:**
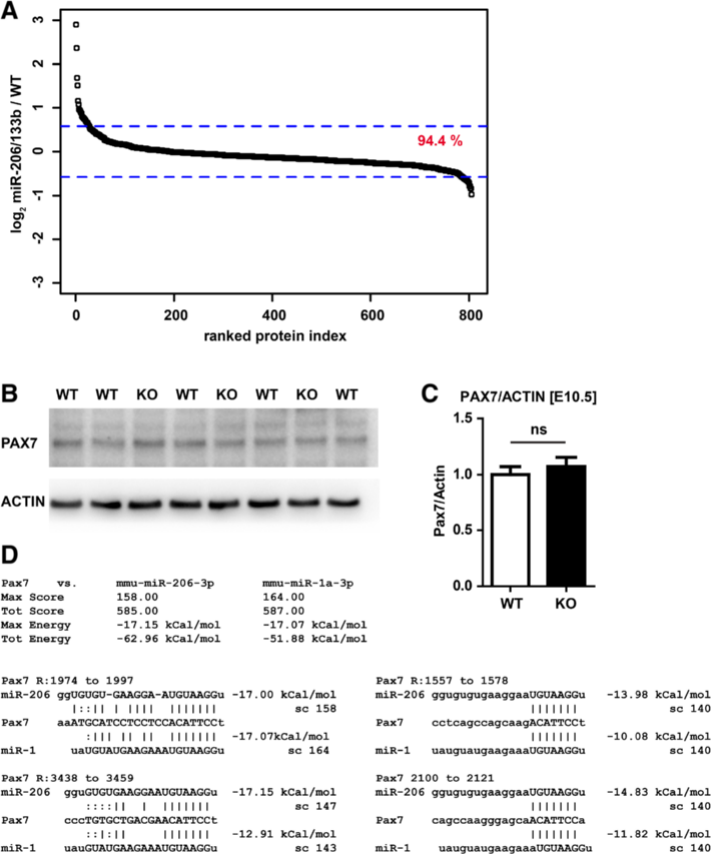
**miR-206/133b does not modulate abundance of PAX7 in E10.5 embryos.** SILAC-based screening of changes in protein abundance based on two independent pairs of miR-206/133b knock-out / wildtype (WT) samples revealed few changes that were greater than 1.5 fold up or down in miR-206/133b knock-out versus WT M. soleus **(A)**. Not one of the proteins expected to be potentially upregulated was a predicted target of miR-206 or miR-133b (microRNA.org). In accordance with transcriptome data, no change in protein abundance could be confirmed in western blot analysis. Loss of miR-206/133b does not change the abundance of PAX7 protein in the trunk of E10.5 embryos **(B,** **C)**. Analysis of the 3′ untranslated region (UTR) of Pax7 using miRanda indicates identical binding sites for miR-206 and miR-1. Differences in the primary sequence of miR-206 and miR-1 do not lead to substantial changes in the predicted miRNA-Pax7 UTR interaction **(D)**.

Since microRNAs regulate expression on the transcript or protein level [26,27], we performed an unbiased SILAC based proteomics screen to detect potential miRNA targets that are regulated at the translational level. Proteins were isolated from M. soleus of WT and miR-206/133b mutant mice and combined with proteins obtained from soleus muscles of ^13^C_6_Lys-labled mice, which serve as a reference for relative quantification. In total, we identified 1,500 proteins and 805 were quantified in two independent experiments. SILAC ratios of WT/^13^C_6_-Lys-WT and in knock-out/^13^C_6_-Lys-WT were used to calculate the direct KO/WT ratios for each of the experiments [19,21]. In accordance with our transcriptome analysis, only few changes in protein concentrations between knock-out and WT soleus muscle were observed (Figure 6A). Although we quantified 94 potential miR-206 targets that displayed a good score according to the miRanda [28] based microRNA.org target prediction database (August 2010 release) [29], none of the potential target protein was upregulated more than 1.5 fold in the knock-out tissue. Likewise, none of the 57 proteins found in our SILAC data set that were predicted as miR-133b targets according to the microRNA.org database were upregulated.

Although no alterations in skeletal muscle development of miR-206/133b mutants were apparent, we investigated the potential regulatory interaction between miR-206 and Pax7 in somites of E10.5 embryos. No changes in Pax7 protein expression were detected in mutant compared to WT embryos indicating that miR-206 is not required to suppress expression of Pax7 in somites (Figure 6B, C), probably due to the presence of miR-1, which shows a similar expression pattern as miR-206 during skeletal muscle development but is more widely expressed compared to miR-206 in adult muscles. In fact, the seed sequences of miR-1 and miR-206 are identical, which predicts a comparable repression of Pax7 by either miR-206 or miR-1 (Figure 6D) according to target algorithms like miRanda [28]. Taken together, neither transcriptome nor proteome analysis revealed significant changes in gene expression between WT and KO mice, which is in line with the lack of a muscle specific phenotype in miR-206/133b knock-out mice.

### Loss of the miR-206/133 cluster does not affect skeletal muscle regeneration in Duchenne muscular dystrophy model mice

**Figure 7:**
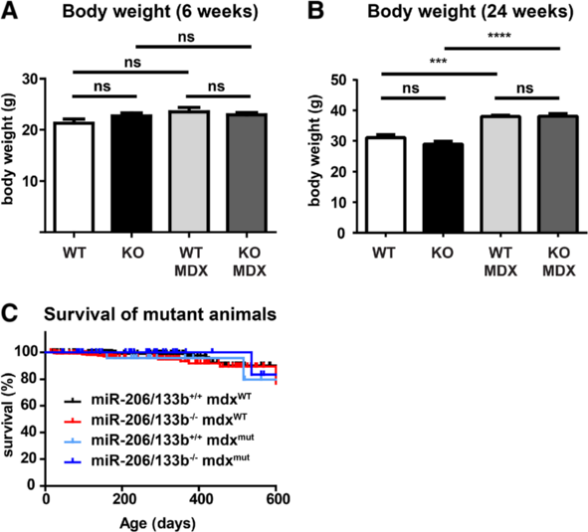
**No difference in body weight and survival of miR-206/133b and mdx mutant mice. (A)** The body weight of male animals is not different at 6 weeks of age (n ≥11/group); however, at later ages **(B)**, the *mdx* mutation leads to an increase in body weight that is not further modified by mutation of the miRNA cluster (wildtype (WT): miR-206/133b^+/+^, knock-out (KO): miR-206/133b^−/−^; MDX: mdx^Y/-^; n ≥5/group). **(C)** Survival of miR-206/133b mutants is not impaired. The *mdx* mutation does not affect survival of miR-206/133b knock-out mice.

**Figure 8:**
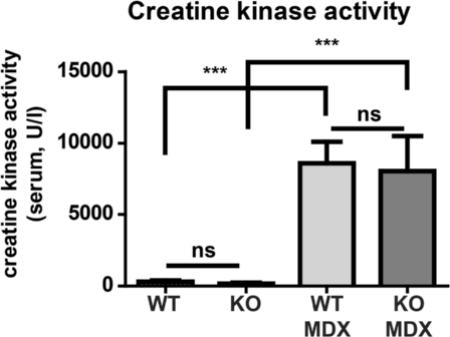
**Loss of miR-206/133b does not change serum creatine kinase activity.** Creatine kinase activity in the serum in miR-206/133b mice knock-out mice is not altered. The *mdx* mutation leads to strong increase of creatine kinase activity. No differences were observed in *mdx* versus miR-206/133b KO / MDX mice (n ≥6/group).

**Figure 9:**
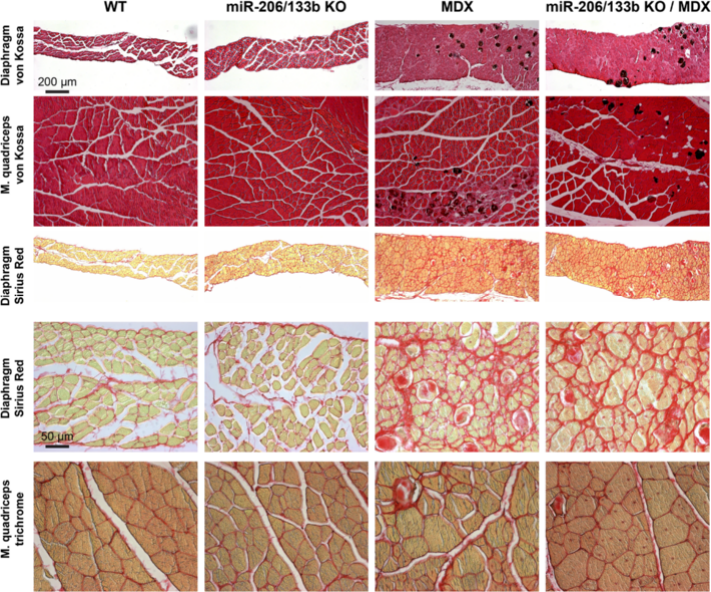
**miR-206/133b mutation does not modulate muscle loss or regeneration in Mdx mice.** No histological changes are observed in diaphragm or M. quadriceps after loss of miR-206/133b. Mutation of the mouse dystrophin gene (MDX) leads to increased muscle mass, calcifications (von Kossa staining) and increased fibrosis (red signal in Sirius red staining). Additional loss of miR-206/133b does not lead to changes of the *mdx* phenotype. Sections obtained from 12 weeks old animals are shown, similar results are obtained using tissue from 6-week- and 9-month-old mice. The scale bar corresponds to 200 μm in the upper three rows and to 50 μm in the lower two rows.

Previously, miR-206 has been implicated in muscle fiber stability and regeneration of skeletal muscles, although the miR-206 seems dispensable for specification, proliferation and differentiation of skeletal muscle [12]. To analyze the potential function of miR-206/133b miRNAs and their targets in maintenance and regeneration of muscle fibers we generated miR-206/133b^−/−^//mdx^Y/-^ compound mutant mice. *Mdx* mice carry a point mutation in the dystrophin gene on the X-chromosome leading to constant damage and loss of muscle fibers, which is compensated by increased regeneration [30]. miR-206/133b^−/−^/mdx mutant mice were born at the expected Mendelian ratio (Table 1) and developed normally, which is in contrast to the previously described miR-206^−/−^/mdx mutant mice [12]. Body weight development (Figure 7A, B) and survival (Figure 7C) of compound mutant animals was indistinguishable from *mdx* mice. Regeneration of skeletal muscles in mdx mice leads to increased expression of the miR-206/133b cluster in muscles with a low content of type I muscle fibers that express only low levels of miR-206/133b under normal conditions (Figure 3A, [12]). In contrast we observed a moderate, not significant reduction of miR-1 in some mdx muscles (Figure 3B), possibly related to the emergence of other non-muscle cell types in the dystrophic muscles. Hence, it seems possible that such muscle might be affected by inactivation of miR-206/133b in conditions of constant regeneration. Loss of dystrophin expression results in the release of muscle creatine kinase (MCK) from muscle fibers into the blood, which serves as an indicator of muscle injury or muscle degenerative processes [31]. To access the degree of muscle damage in miR-206/133b^−/−^/mdx mutant mice we determined MCK activity in serum of adult mice. Only very low MCK activity was detected in WT and miR-206/133b mice, which did not differ significantly between WT (297 ± 85 U/l, n = 6) and mutants (152 ± 62 U/l, n = 11). Importantly, we observed much higher MCK activities in *mdx* mice (8590 ± 1507 U/l, n = 6), which were not increased any further in miR-206/133b/mdx mutants (8024 ± 2464 U/l, n = 6; Figure 8) indicating that compound mutants do not suffer from increased muscle fiber damage compared to *mdx* mice. Next, we performed a comprehensive histological analysis of different muscles of the hind limb, as well as of the diaphragm from WT, miR-206/133b^−/−^, mdx^Y/-^ and miR-206/133b^−/−^//mdx^Y/-^ mice. Trichrome- and Sirius Red-, and von Kossa staining were used to determine myofiber organization, fibrosis, and intramuscular calcifications often present in mdx mice (Figure 9). No differences were found between WT and miR-206/133b skeletal muscle based on Trichrome- (not shown), von Kossa or Sirius red staining including absence of fibrosis and calcifications. Mutation of dystrophin caused the typical signs of muscle dystrophy, namely massive thickening of the diaphragm, enhanced fibrosis and patches of calcified tissue within the diaphragm and the M. quadriceps (Figure 9). No calcified fibers were observed in the soleus muscle. Deletion of miR-206/133b in *mdx* mice did not cause a more severe phenotype in the soleus, quadriceps or diaphragm muscles compared to single mutant *mdx* mice. Together these data indicate that loss of miR-206/133b cluster does not increase muscle damage in mdx mice or compromise skeletal muscle regeneration in constantly renewing muscle tissue.

**Figure 10:**
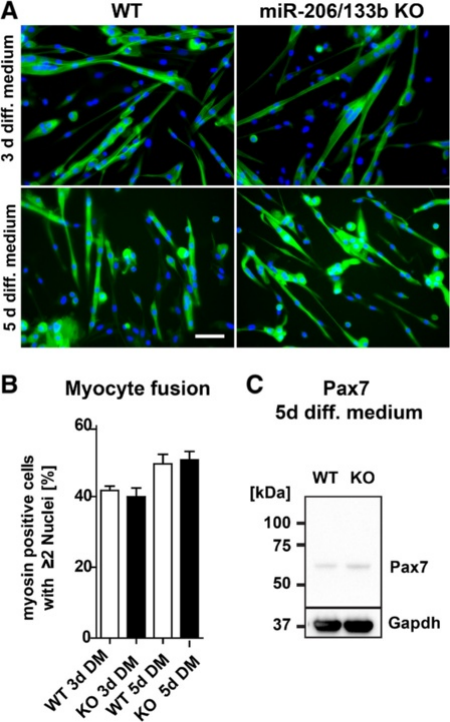
**No differences in differentiation of muscle progenitor cells** ***in vitro*** **.** Proliferating muscle stem cells isolated from hind limb muscles of wildtype (WT) and miR-206/133b knock-out mice (KO) were induced to differentiate and stained after 3 or 5 days in differentiation medium (DM) for expression of myosin (green: MF20 staining, blue: nuclear staining; the scale bar corresponds to 50 μm). No differences in cell morphology or differences in the expression of myosin were detected between WT and mutant cells **(A)**. Formation of fused myotubes from differentiating myoblasts was not impaired by the loss of the miR-206/133b cluster **(B)**. Pax7 expression in differentiating myogenic progenitor cells was detected by western blot. No differences in Pax7 expression were detected after loss of miR-206/133b **(C)**.

To further analyze a possible function of the miR-206/133b cluster in the differentiation of muscle stem cells, we isolated muscle satellite cells from WT and miR-206/133b mutant mice. Loss of the miR-206/133b cluster did not impair the ability of muscle stem cells to differentiate to myotubes *in vitro* (Figure 10A). We did not observe reduced differentiation as accessed by the number of MF20 positive cells nor changes in the fusion of myocytes as accessed by the number of myotubes with more than one nucleus (Figure 10B). Moreover, we found that the lack of miR-206/133b did not increase the concentration of the potential miR-206 and miR-1 target Pax7 in differentiating satellite cells on the 5th day after induction of differentiation (Figure 10C).

## Discussion

Physiological properties of skeletal muscle cells including specification, differentiation, function and regeneration are controlled by an extensive network of regulatory molecules [32]. Many key genes have been identified that are essential for specification and differentiation of muscle precursor cells during development. Commitment of muscle cells is controlled by a core program of myogenic regulatory factors of the MyoD family, which also control transcription of the miR-1/206/133 miRNA clusters [6,33] resulting in a prominent expression of these miRNAs in muscle cells. Yet, important differences in the expression pattern of miR-1/206/133 miRNA clusters exist: the miR-1/133a clusters are expressed in heart and all skeletal muscles, while expression of miR-206/133b becomes restricted to a subset of oxidative muscle fibers by hitherto unknown molecular mechanisms. Since the two miR-1/133a clusters display virtually identical expression patterns, partially overlapping or redundant functions of these miRNAs seem likely. In contrast, the miR-206/133b cluster shows a more restricted expression, which might argue for a distinct function of this cluster albeit muscle cells expressing miR-206/133b also transcribe miR-1/133a. In addition, the primary sequence of miR-206 differs from miR-1 while miR-133b is very similar to miR-133a. However, the seed sequences of miR-206 and miR-1 important for target recognition [1] are identical, indicating only limited differences in the target specificity of miR-206 and miR-1. The miR-206/133b locus also directs expression of the long non-coding RNA linc-MD1, which was postulated to act as a competing endogenous RNA or miRNA decoy [9] adding a further potential function to the miR-206/133b locus.

We have generated a miR-206/133b knock-out allele by deletion of the genomic region spanning from miR-206 to miR-133b and resulting in removal of the third exon of the linc-MD1 that might act as a miR-133 sponge. Surprisingly, homozygous deletion of the miR-206/133b locus did not cause an apparent phenotype in adult mice. No changes in the morphology and distribution of muscle fiber types were apparent and the transcriptional and proteomics signatures of miR-206/133b mutant muscles were virtually unchanged, ruling out that miR-206 and/or 133b alone play an essential role in muscle physiology. These observations are consistent with to observations made in miR-206 knock-out mice, which also show no obvious phenotype under baseline conditions [12]. Apparently, the concomitant loss of miR-206, miR-133b and the potential miR-133 sponge in linc-MD1 is compatible with normal muscle development and functions, which might be explained by expression of the miR-1/133a clusters in type I myofibers compensating for deletion of miR-206/133b. Theoretically, the inactivation of miR-133b might also be counteracted by the loss of the miR-133 sponge function, although the physiological relevance of endogenous competing RNAs has been questioned making this explanation less likely [34].

We found a more widespread and abundant expression of the miR-206/133b cluster in regenerating muscles, which confirms previous findings [12]. It seems possible that the expression of miR-206 in regenerating muscle reflects a specific function of the miR-206/133b in the regenerative response. However, it is important to point out that miR-206/133b is broadly expressed during development in immature muscle cells. Hence, it is difficult to exclude that the increased expression of miR-206/133b during skeletal muscle regeneration simply mirrors the immature state of newly formed myofibers that have not yet terminated expression of the miR-206/133b cluster in the majority of mature type II fibers [35]. The lack of a more severe phenotype in miR-206/133b^−/−^//mdx^Y/-^ compound mutants compared to mdx^Y/-^ mice clearly argues against an important role of the miR-206/133b cluster for skeletal muscle regeneration. Moreover, we did not see an increase in lethality or an increase in serum creatine kinase levels in compound mutant mice, which also excludes an important role of the miR-206/133b cluster in restricting myofiber damage.

miR-206 has been claimed to suppress Pax7 expression thereby regulating muscle stem cell renewal and differentiation. The normal expression of Pax7 in isolated miR-206/133b mutant satellite cells and the normal differentiation of mutant satellite cells to myotubes *in vitro* do not argue for an important role of miR-206 in Pax7 expression. These results are in contrast to previous publications mostly relying on the inhibition of miR-206 by anti-miRs [16], overexpression of miR-206 [15,36], or by correlation of expression profiles [37,38]. It seems possible that previous anti-miR approaches were biased by off-target effects or affected expression of miR-1, which can also suppress Pax7. Generation of skeletal muscle specific miR-1/miR133a//miR-206/133b triple mutants will probably solve this controversy in the future. We do not have a convincing explanation for the attenuation of Pax7 down-regulation in differentiating miR-206-deficient satellite cells reported by Liu *et al*. [12]. The same authors also described that miR-206/mdx mutant mice suffer from increased muscle degeneration, impaired regeneration and premature death, which was all not seen in our miR-206/133b mutants. In principle, it is feasible that the concomitant loss of mir-206 and miR-133b in our model accounts for these differences, which would strengthen the notion that the miR-206/133b cluster acts as a functional unit targeting similar biological processes sometimes in parallel and sometimes in opposing directions [2,11]. Alternatively, subtle differences in the genetic background and/or strain-depended differences in the expression of miR-1/133a compensating for the loss of miR-206/133b might differentially affect the course of muscular dystrophy in miR-206/133b^−/−^//mdx^y/-^ and miR-206^−/−^//mdx^y/-^ compound mutants.

## Conclusion

Taken together, our results question a major role of the miR-206/133b cluster in development, function and regeneration of skeletal muscle in mouse and argue for overlapping functions of the miR-206/133b and miR-1/133a gene clusters during myogenesis. Our findings are in line with the hypothesis that individual miRNAs often fine-tune biological processes and play modulatory roles. Further genetic studies targeting the miR-1/133a together with the miR-206/133b gene clusters specifically in skeletal muscle will probably reveal the prevalent physiological function of miR-1/206/133 in mammals.

## Abbreviations

BSA: bovine serum albumin
ChIP: chromatin immunoprecipitation
DM: differentiation medium
DSHB: developmental studies hybridoma bank
DTA: diphtheria toxin A
ES cell: embryonic stem cell
FCS: fetal calf serum
FDR: false discovery rate
HRP: horseradish peroxidase
IRES: internal ribosome entry site
KO: knock-out
lacZ: β-galactosidase coding
MCK: creatine kinase, muscle
MDX: mouse model of Duchenne Muscular Dystrophy, X-linked
miR: miRNA
PBS: phosphate buffered saline
PFA: paraformaldehyde
qRT-PCR: quantitative reverse transcription polymerase chain reaction
SDS-PAGE: sodium dodecyl sulfate polyacrylamide gel electrophoresis
SEM: standard error of the mean
SILAC: stable isotope labeling by amino acids in cell culture
UTR: untranslated region
WT: wildtype
